# IL-37 isoform D acts as an inhibitor of soluble ST2 to boost type 2 immune homeostasis in white adipose tissue

**DOI:** 10.1038/s41420-022-00960-3

**Published:** 2022-04-05

**Authors:** Chaoze Li, Mingsheng Zhao, Ming Zhao, Nuo Chen, Yaxin Guo, Yingxin Du, Yi Zhang, Baihui Cao, Bing Zhan, Chun Guo, Yuan Li, Yan Li, Yongyu Shi, Faliang Zhu, Lining Zhang, Qun Wang

**Affiliations:** 1 Key Laboratory of Infection and Immunity of Shandong Province, Shandong Provincial Clinical Research Center for Immune Diseases and Gout, Department of Immunology, School of Basic Medical Sciences, Cheeloo College of Medicine, Shandong University, Jinan, Shandong 250012 China; 2 Department of pathogenic biology, School of Basic Medical Sciences, Cheeloo College of Medicine, Shandong University, Jinan, Shandong 250012 China

**Keywords:** Obesity, Cytokines, Inflammation

## Abstract

White adipose tissue (WAT) homeostasis substantiated by type 2 immunity is indispensable to counteract obesity and metabolic disorders. IL-33/suppression of tumorigenicity (ST) 2 signaling promotes type 2 response in WAT, while potential regulators remain to be discovered. We identified human IL-37 isoform D (IL-37D) as an effective trigger for ST2-mediated type 2 immune homeostasis in WAT. IL-37D transgene amplified ST2^+^ immune cells, promoted M2 macrophage polarization and type 2 cytokine secretion in WAT that mediate beiging and inflammation resolution, thereby increasing energy expenditure, reducing obesity and insulin resistance in high-fat diet (HFD)-fed mice. Mechanistically, either endogenous or exogenous IL-37D inhibited soluble ST2 (sST2) production from WAT challenged with HFD or TNF-α. Recombinant sST2 impaired the beneficial effects of IL-37D transgene in HFD-fed mice, characterized by damaged weight loss, insulin action, and type 2 cytokine secretion from WAT. In adipose-derived stem cells, IL-37D inhibited TNF-α-stimulated sST2 expression through IL-1 receptor 8 (IL-1R8)-dependent NF-κB inactivation. Collectively, human IL-37D suppresses sST2 to boost type 2 immune homeostasis in WAT, which may be a promising therapy target for obesity and metabolic disorders.

## Introduction

Obesity increases the risk for many life-threatening diseases including type 2 diabetes mellitus, cardiovascular diseases, and cancer [1–3]. Excess fat accumulation in white adipose tissue (WAT) can induce metabolic inflammation and insulin resistance, which in turn aggravate obesity development. During this process, WAT goes through a switch from immune homeostasis to inflammation. In lean WAT, type 2 immune cells composed of alternatively activated M2 macrophages, group 2 innate lymphoid cells (ILC2s), eosinophils and regulatory T cells (Tregs), and so on, maintain immune homeostasis of WAT. While in obese WAT, amounts of type 1 immune cells including classically activated M1 macrophages, effector helper T (Th) 1 cells, and cytotoxic T lymphocytes are accumulated to mediate metabolic inflammation [4–6]. In addition to maintaining WAT immune homeostasis in lean condition, type 2 immune circuit plays critical role in limiting type 1 inflammation and driving WAT beiging to fight obesity and associated metabolic disorders.

Interleukin (IL)-33/suppression of tumorigenicity (ST) 2 signaling plays a critical role in maintaining type 2 immune homeostasis in WAT. Upon binding the membrane receptor ST2, IL-33 stimulates the expansion or activation of different kinds of type 2 immune cells including ILC2s, Tregs, and M2 macrophages to support type 2 responses characterized by type 2 cytokines like IL-4, IL-5, IL-10, and IL-13, thereby providing protection against obesity and insulin resistance [5, 7–12]. In response to high-fat diet (HFD) challenge, mice deficient in IL-33 or ST2 showed increases in obese phenotype and insulin resistance [13, 14], suggesting the essential role of IL-33/ST2 signaling in reducing obesity and related complications. In contrast to the full-length ST2, soluble ST2 (sST2) in a secretory form lacks the transmembrane and cytoplasmic domains and acts as a decoy receptor for IL-33 to block IL-33/ST2 signaling [15, 16]. Very recently, sST2 is verified as an adipocyte-secreted adipokine to disrupt WAT Treg/ILC2 homeostasis, thereby aggravating obesity-associated insulin resistance in mice [17]. It can be seen that sST2 may serve as a promising target for obesity treatment, by which type 2 immune homeostasis can be reinforced in WAT, while more effective molecules or approaches regulating sST2-ST2 balance remain to be determined.

IL-37 is a new member of IL-1 family presented in human but not mice. It consists of different isoforms A, B, C, D, E, each one is believed to be expressed in a tissue-specific manner, despite that their detailed expression profiles remain to be uncovered [18–22]. Among them, IL-37 isoform B (IL-37B) composed of exons 1, 2, 4–6 is the largest one and has a constitutive or inducible expression in innate immune cells to exert anti-inflammatory effect. Several studies have demonstrated that human IL-37B protects against lipopolysaccharide (LPS)-induced endotoxemia, dextran sulfate sodium (DSS)-induced colitis, and obesity-induced inflammation [20–26]. Differently, the isoform D of IL-37 (IL-37D) is encoded by exons 1, 4-6 and may have its unique expression profile in tissues or cells. Using human IL-37D-transgenic (IL-37Dtg) mouse model, we showed that IL-37D alleviated endotoxemia or colitis through suppressing the production of IL-1β, IL-6, and TNF-α or inhibiting the expression of NLRP3 in macrophages [27, 28]. Our previous study and several GEO profiles have shown the dominant expression of IL-37D in human adipose tissue and adipose tissue-derived stem cells (ADSCs) [27], whereas its exact roles in WAT and possible influences on obesity remain to be clarified. Despite that IL-37D possesses some functions similar to IL-37B, such as negative regulation of innate immunity, it remains to be clarified whether IL-37D regulates IL-33/ST2 signaling and type 2 immunity, particularly in WAT.

In the present study, using IL-37Dtg mice in combination with recombinant sST2 protein, we showed that IL-37D attenuated diet-induced obesity, insulin resistance, and hepatic steatosis by promoting type 2 response to reinforce WAT immune homeostasis and increase energy expenditure, while recombinant sST2 impaired the beneficial effects of IL-37D in HFD-fed mice. Either endogenous or exogenous IL-37D blocked sST2 production from WAT or ADSCs, which was dependent on IL-1 receptor 8 (IL-1R8)-dependent NF-κB inactivation. Thus, IL-37D plays a critical role in suppressing sST2 in WAT, which promotes type 2 immunity via ST2^+^ immune cell amplification, M2 macrophage polarization, and type 2 cytokine secretion, thereby inducing WAT beiging and inflammation resolution.

## Results

### IL-37D transgene attenuates obesity, insulin resistance, and hepatic steatosis in HFD-fed mice

**Fig. 1:**
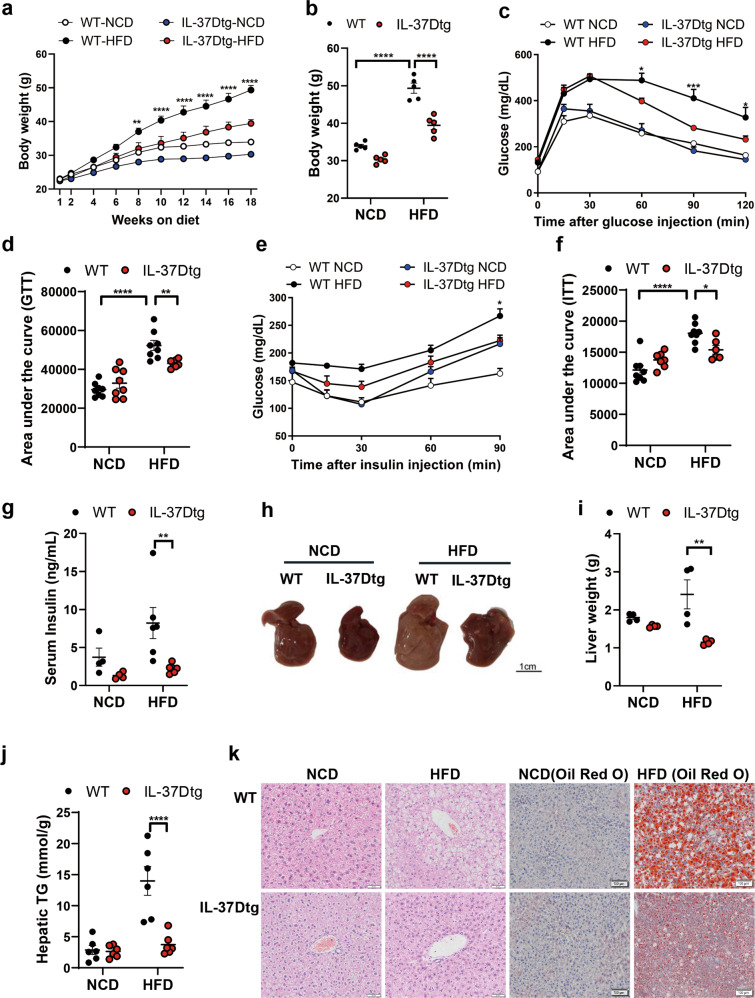
IL-37D transgene attenuates obesity, insulin resistance, and hepatic steatosis in HFD-fed mice. IL-37Dtg and WT mice were administrated with HFD or NCD for 18 or 23 weeks (*n* = 4–8 per group). Body weight recorded during (**a**) and after (**b**) 18 weeks of diet intervention. Glucose tolerance test (GTT, **c**) and insulin tolerance test (ITT, **e**) were performed in mice after 11 or 13 weeks of HFD, respectively, the values of area under the curve in GTT (**d**) and ITT (**f**) were measured. After 18 or 23 weeks of HFD, serum insulin (**g**) was detected; the morphology (**h**), weight (**i**), and triglyceride (TG) contents (**j**) of liver tissues are shown; the hematoxylin & eosin (H&E) and Oil Red O staining were performed in liver sections (**k**). Data represent mean ± SEM. *p < 0.05, **p < 0.01, ***p < 0.001, ****p < 0.0001, determined by two-way ANOVA (**a**–**g**, **i–j**).

To explore the possible influences of human IL-37D on obesity and related metabolic disorders, we used IL-37Dtg and wild type (WT) mice to establish HFD-induced obesity. In IL-37Dtg mice, human IL-37D mRNA was verified to be ectopically expressed in the metabolic tissues including liver, adipose tissues, and skeletal muscle (Fig. S1). With the extension of HFD feeding time, these mice showed significant decrease in body weight compared with WT mice (Fig. 1a, b). In line with the change of body weight, HFD-fed IL-37Dtg mice showed an obvious improvement in glucose tolerance and insulin sensitivity compared with HFD-fed WT mice, as suggested by significant decreases in glucose levels after glucose or insulin injection and total declines in values of area under the curve (Fig. 1c–f). Meanwhile, the elevation of serum insulin caused by HFD was reversed to a relatively normal level by IL-37D transgene (Fig. 1g). Next, we examined the influences of IL-37D transgene on fatty liver in HFD-fed mice. The results showed that long-term of HFD induced obvious hepatic steatosis in WT mice rather than in IL-37Dtg mice. The livers from HFD-fed IL-37Dtg mice displayed smaller and darker morphology, particularly lower weights and triglyceride (TG) contents than those from HFD-fed WT mice (Fig. 1h–j). Accordingly, the liver sections from HFD-fed IL-37Dtg mice showed obviously smaller lipid droplets than those from HFD-fed WT mice (Fig. 1k). These observations suggest that IL-37D transgene protects the mice against HFD-induced obesity, insulin resistance, and hepatic steatosis.

### IL-37D transgene promotes energy expenditure through thermogenesis in HFD-fed mice

**Fig. 2:**
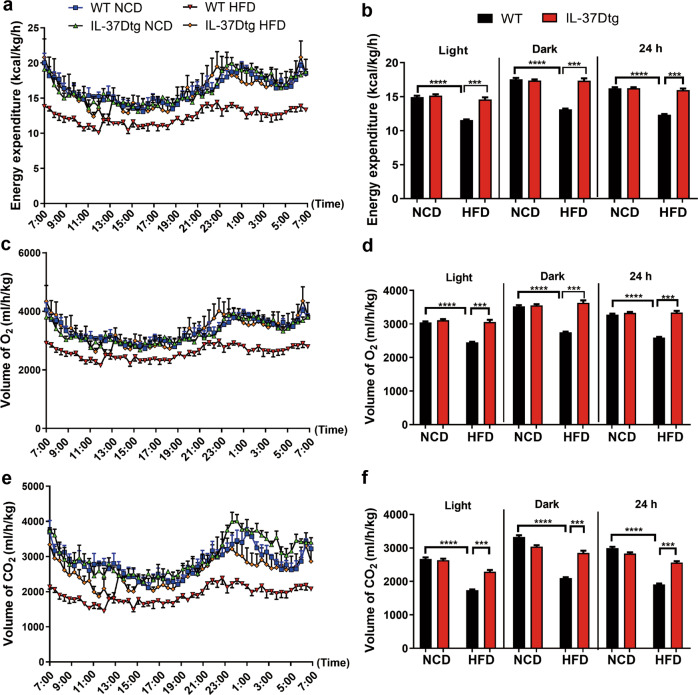
IL-37D transgene increases energy expenditure in HFD-fed mice. Male IL-37Dtg and WT mice were fed on HFD or NCD (*n* = 4 per group). The energy expenditure (**a**, **b**), VCO_2_ (**c**, **d**), and VO_2_ (**e**, **f**) were measured in individual mouse using calorimetry system. Data represent mean ± SEM. ***p < 0.001, determined by two-way ANOVA (**b**, **d**, **f**).

To delineate the direct reasons for the reduction of HFD-induced obesity in IL-37Dtg mice, we further measured energy expenditure, locomotory activity, and food intake in these mice. Compared with normal chow diet (NCD) feeding, HFD feeding led to significant decrease of energy expenditure in WT mice, but failed to cause decrease in IL-37Dtg mice under both light and dark cycles (Fig. 2a, b). In response to HFD, IL-37Dtg mice consumed more O_2_ and generated more CO_2_ than WT mice, demonstrating that IL-37D transgene promotes energy expenditure via thermogenesis dependent on oxidative metabolism in HFD-fed mice (Fig. 2c–f). Furthermore, no differences were detected in locomotor activity and diet intake between IL-37Dtg and WT mice under either NCD or HFD conditions (Fig. S2a–c). These findings suggest that IL-37D reduces obesity through increasing energy expenditure other than influencing diet intake or locomotor activity in HFD-fed mice.

### IL-37D transgene promotes WAT homeostasis and beiging via upregulating type 2 cytokines in HFD-fed mice

**Fig. 3:**
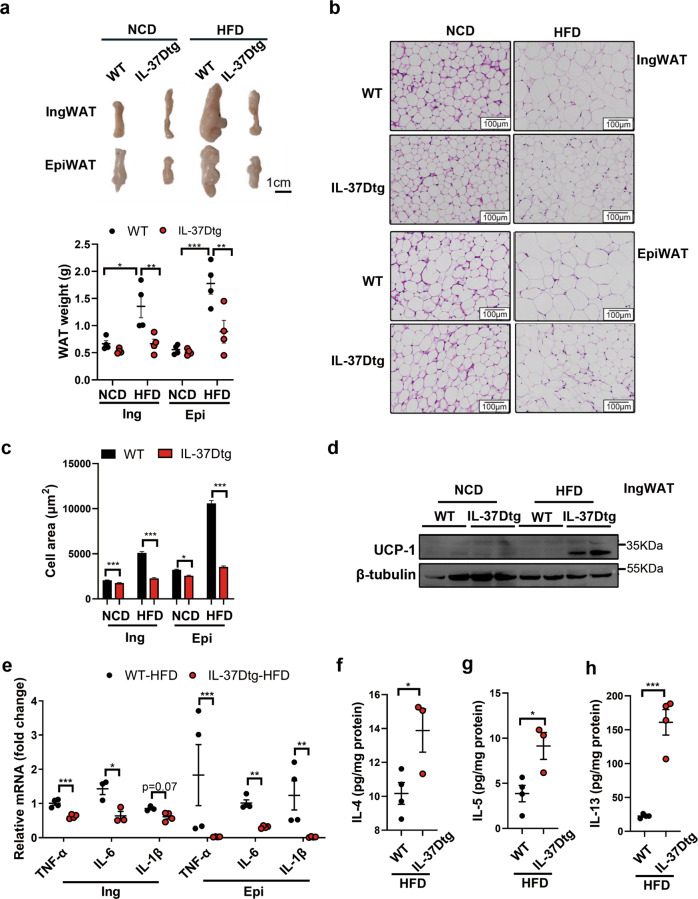
IL-37D transgene promotes WAT homeostasis and beiging via upregulating type 2 cytokines in HFD-fed mice. **a**–**d** IL-37Dtg and WT mice were fed on 18 weeks of HFD (*n* = 4 per group). The morphology and weight (**a**) of inguinal and epidydimal WAT (IngWAT, EpiWAT) are shown. WAT sections were stained with H&E (**b**) and adipocyte sizes were measured using Image-J (**c**). The protein levels of UCP-1 (**d**) were detected by western blot. **e**–**h** IL-37Dtg and WT mice fed on 23 weeks of HFD (*n* = 4 per group). The mRNA levels of IL-6, IL-1β, and TNF-α in WAT explants were determined by qPCR (**e**). The secretion levels of IL-4 (**f**) IL-5 (**g**) and IL-13 (**h**) from inguinal WAT explants were detected by ELISA. Data represent mean ± SEM. *p < 0.05, **p < 0.01, ***p < 0.001, determined by two-way ANOVA (**a**, **c**) or student’s *t* test (**e**–**h**).

Considering the critical roles of WAT in regulating energy homeostasis, we next examined the effects of IL-37D transgene on WAT homeostasis and beiging in HFD-fed mice. In line with its effect on weight loss, IL-37D transgene caused significant reductions in the weights of both inguinal (Ing) and epidydimal (Epi) WAT in HFD-fed mice (Fig. 3a). For both inguinal and epididymal WAT, adipocyte hypertrophy caused by HFD was obviously relieved by IL-37D transgene, evidenced by significant decreases in adipocyte sizes. Even under NCD condition, adipocyte sizes in these fat pads showed slight decreases in IL-37Dtg mice compared with WT mice (Fig. 3b, c). Importantly, UCP-1 protein was easily detected in inguinal WAT from HFD-fed IL-37Dtg mice compared with that from HFD-fed WT mice (Fig. 3d), indicating that IL-37D transgene could promote WAT beiging to dissipate energy in HFD-fed mice. Furthermore, IL-37D transgene also caused a decrease of brown adipose tissue (BAT) weight in HFD-fed mice, together with small and multilocular lipid droplets inside brown adipocytes and increased UCP-1 protein in BAT (Fig.S3a–d), indicating that BAT activation also contributes to energy dissipation in IL-37Dtg mice when challenged with HFD.

On the other hand, the mRNA levels of TNF-α, IL-6, and IL-1β in WAT, especially in epidydimal WAT, were obviously decreased by IL-37D transgene in HFD-fed mice; whilst the secretion levels of TNF-α from both WAT explants were significantly declined by IL-37D transgene in these mice (Fig. 3e; Fig. S4a). These data demonstrate that IL-37D transgene could reduce WAT inflammation in HFD-fed mice. Since type 2 cytokine-mediated responses exert direct or indirect roles in limiting type 1 inflammation or promoting beiging in WAT, it is possible that IL-37D transgene could affect type 2 response in WAT. We, therefore, examined the levels of type 2 cytokines secreted from WAT explants of HFD-fed mice. As expected, the secretion of type 2 cytokines IL-4, IL-5, and IL-13 from WAT were significantly increased by IL-37D transgene in mice fed with 23 weeks of HFD (Fig. 3f–h; Fig. S4b). Thus, IL-37D may play a crucial role in enhancing type 2 cytokine-mediated response to maintain WAT homeostasis in response to HFD.

### IL-37D transgene amplifies ST2^+^ immune cell population and promotes M2 macrophage polarization in WAT via sST2 suppression in HFD-fed mice

**Fig. 4:**
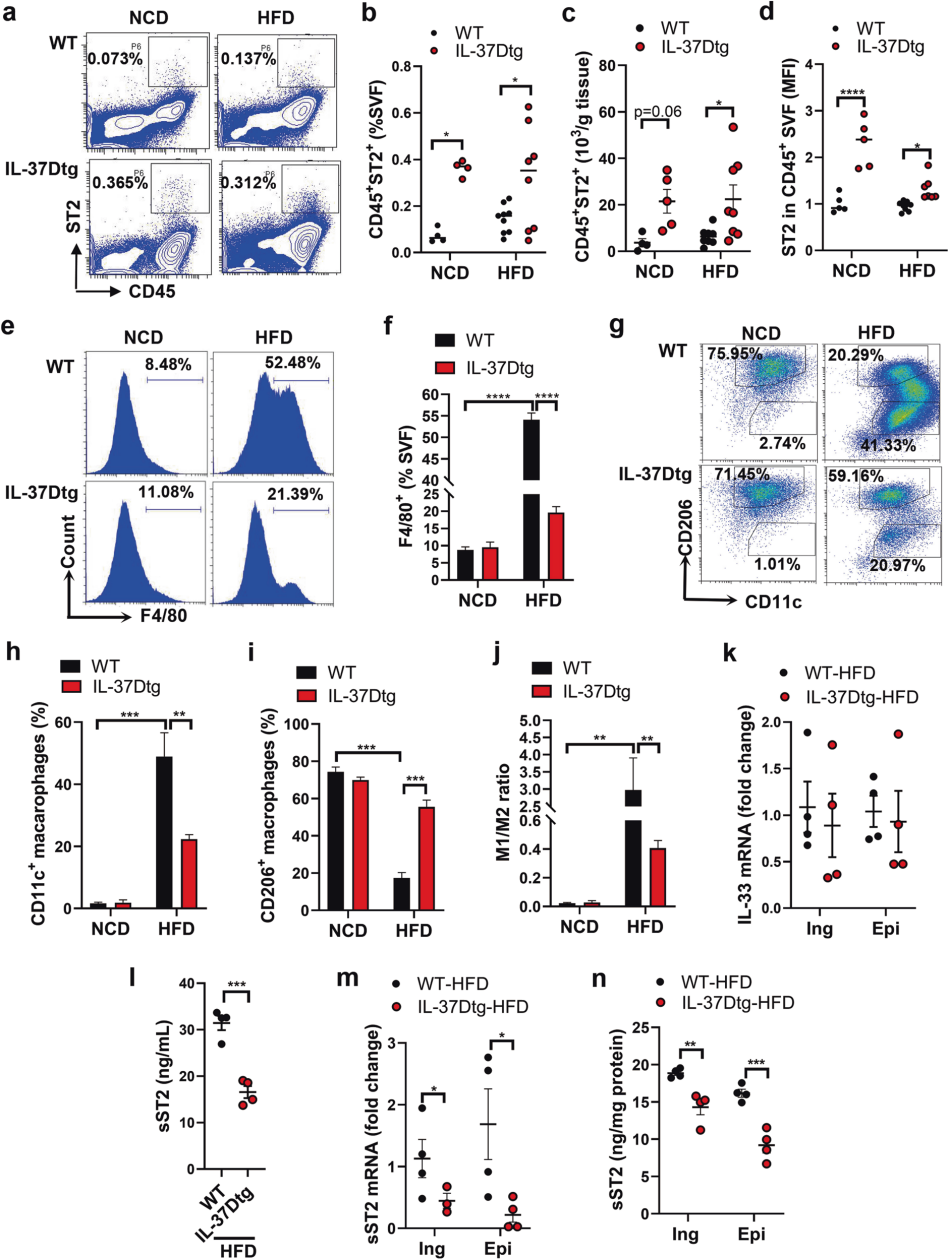
IL-37D transgene amplifies ST2^+^ immune cell population and promotes M2 macrophage polarization in WAT via sST2 suppression in HFD-fed mice. IL-37Dtg and WT mice were fed on HFD or NCD for 18 weeks (*n* = 4–8 per group). **a**–**d** SVF from inguinal WAT was analyzed by flow cytometry. Representative (**a**) and statistical data on the percentages of CD45^+^ST2^+^ cells (**b**), the numbers of CD45^+^ST2^+^ cells (**c**), and the mean fluorescence intensity (MFI) of ST2 (**d**) are shown. **e**–**j** SVF from epidydimal WAT was analyzed by flow cytometry. Representative (**e**) and statistical (**f**) data on the percentages of F4/80^+^ macrophages are shown. Representative (**g**) and statistical (**h**, **i**) data on the percentages of CD11c^+^ M1 macrophages and CD260^+^ M2 macrophages in F4/80^+^ cells are shown. The ratio of M1 to M2 (**j**) was calculated. **k**–**n** The mRNA levels of IL-33 in WAT were determined by qPCR (**k**). The serum level of sST2 (**l**) was measured by ELISA. The mRNA (**m**) and secretion (**n**) levels of sST2 in WAT explants were determined by qPCR and ELISA. Data represent mean ± SEM. *p < 0.05, **p < 0.01, ***p < 0.001, ****p < 0.0001 determined by two-way ANOVA (**b**, **c**, **d**, **f**, **h**–**j**) or student’s *t* test (**k**–**n**).

IL-33/ST2 signaling is critical for the maintenance of type 2 immune homeostasis in WAT. In order to clarify the contributions of type 2 immune cells to IL-37D-mediated WAT homeostasis, we determined the population of CD45^+^ST2^+^ immune cells responsive to IL-33/ST2 signaling in stromal vascular fraction cells (SVF) from inguinal WAT by flow cytometry. The results showed that the percentage of CD45^+^ST2^+^ cells in SVF and their total number in WAT were significantly increased by IL-37D transgene in HFD-fed mice, to some extent, even in NCD-fed mice; the expression level of ST2 in CD45^+^ immune cells was accordingly elevated in these mice (Fig. 4a–d). These observations demonstrate that IL-37D could enhance IL-33/ST2 signaling to amplify ST2^+^ immune cells in WAT, thereby promoting type 2 response-mediated WAT homeostasis in HFD-fed mice. As type 2 cytokines like IL-4 plus IL-13 can stimulate M2 macrophage polarization, which functions in both inflammation resolution and beiging in WAT, we further examined the infiltration and polarization of macrophages in SVF of epidydimal WAT. As expected, HFD feeding remarkably upregulated the percentage of F4/80^+^ macrophages in WT mice, whereas this upregulation was heavily discounted in HFD-fed IL-37Dtg mice (Fig. 4e, f). More importantly, among the F4/80^+^ macrophages, the increase of CD11c^+^ M1 macrophages caused by HFD feeding was remarkably attenuated by IL-37D transgene; whilst the decrease of CD206^+^ M2 macrophages was successfully reversed to a higher level, leading to a reduction of M1 to M2 ratio in HFD-fed IL-37Dtg mice compared with that in HFD-fed WT mice (Fig. 4g–j). Thus, IL-37D transgene could not only reduce HFD-induced macrophage infiltration in WAT, but also skew the macrophage polarization toward M2 phenotype.

Since ST2 signaling is promoted by IL-33 but inhibited by sST2, we further checked their expression in WAT of these mice. Unexpectedly, there was no difference in the mRNA level of IL-33 in WAT between WT and IL-37Dtg mice fed on HFD (Fig. 4k). But to our surprise, a remarkable decrease of serum level of sST2 was observed in HFD-fed IL-37Dtg mice compared with WT mice (Fig. 4l). Considering sST2 is recently recognized as an adipokine from WAT, we thereafter measured the expression and production of sST2 from WAT explants. We showed that IL-37D transgene remarkably downregulated the mRNA and secretion levels of sST2 in either inguinal or epidydimal WAT from HFD-fed mice (Fig. 4m, n). Thus, IL-37D transgene in HFD-fed mice could inhibit the expression and production of sST2 from WAT to enhance ST2 signaling, thereby amplifying ST2^+^ cell population and promoting type 2 immune response in WAT.

### Recombinant sST2 disrupts the protection of IL-37D against obesity, insulin resistance, and hepatic steatosis in HFD-fed mice

**Fig. 5:**
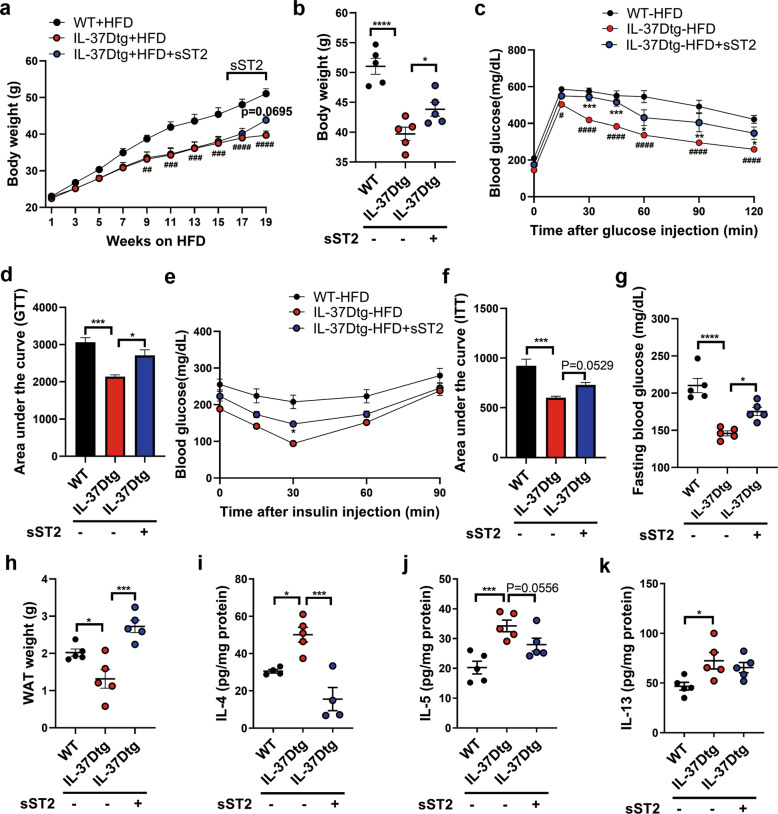
Recombinant sST2 disrupts the protection of IL-37D against obesity, insulin resistance, and hepatic steatosis in HFD-fed mice. IL-37Dtg and WT mice were fed on HFD for 19 weeks (*n* = 5 per group), recombinant sST2 was intraperitoneally injected into IL-37Dtg mice during the last 3 weeks (2 μg every 3 days). Body weight was recorded during (**a**) and after (**b**) the intervention. Glucose tolerance test (GTT, **c**) and insulin tolerance test (ITT, **e**) were performed, respectively, the values of area under the curve in GTT (**d**) and ITT (**f**) were measured. The fasting level of blood glucose was measured (**g**). The weight of inguinal WAT (**h**) was examined and the secretion levels of IL-4 (**i**), IL-5 (**j**), IL-13 (**k**) from its explants were detected by ELISA. Data represent mean ± SEM. *p < 0.05, **p < 0.01, ***p < 0.001, ****p < 0.0001 determined by two-way ANOVA (**a**, **c, e**) or one-way ANOVA (**b**, **d**, **f**–**k**).

To verify whether the beneficial effects of IL-37D transgene on metabolic improvement are dependent on its suppression on sST2, recombinant sST2 was administrated into HFD-fed IL-37Dtg mice to observe the reversal effects on body weight and metabolic parameters. In contrast to the weight loss caused by IL-37D transgene, 3 weeks of sST2 treatment caused mild but significant increase of weight gain in HFD-fed IL-37Dtg mice (Fig. 5a, b). Accordingly, sST2 obviously disrupted the improvement effects of IL-37D transgene on glucose tolerance and insulin sensitivity in HFD-fed mice. Treatment with sST2 caused a marked increase of blood glucose levels in HFD-fed IL-37Dtg mice after glucose injection, while a mild increase after insulin injection (Fig. 5c–f). These mice showed a significant increase in fasting levels of blood glucose compared with non-treated HFD-fed IL-37Dtg mice (Fig. 5g). Particularly, sST2 led to an elevation in inguinal WAT weight in HFD-fed IL-37Dtg mice (Fig. 5h). For mice fed on 19 weeks of HFD, the secretion levels of IL-4, IL-5, and IL-13 from both inguinal and epidydimal WAT explants were markedly increased by IL-37D transgene; while sST2 treatment partially repressed the upregulation of these type 2 cytokines in inguinal WAT and fully suppressed them in epidydimal WAT (Fig. 5i–k; Fig. S5), supporting that IL-37D promotes type 2 response in WAT relying on its suppression on sST2 in HFD-fed mice. Collectively, these findings demonstrate that IL-37D provides protection against obesity and metabolic disorders by inhibiting sST2 production and enhancing type 2 immune homeostasis in WAT.

### IL-37D inhibits sST2 production in IL-1R8-dependent manner

**Fig. 6:**
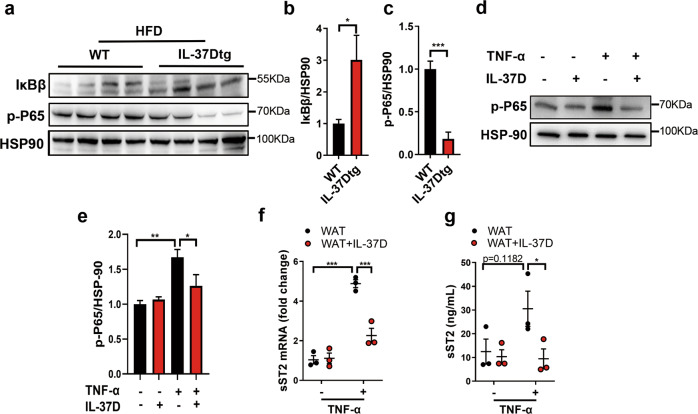
Either endogenous or exogenous IL-37D blocks NF-κB activation and sST2 production in WAT. IL-37Dtg and WT mice were fed on HFD for 23 weeks (*n* = 4 per group). The protein levels of IκB and p-P65 in inguinal WAT were detected by western blot (**a**) and the grayscale values are shown (**b**, **c**). **d**–**g** Epidydimal WAT explants from NCD-fed WT mice were treated with human IL-37D protein (10 ng/mL) in the presence or absence of TNF-α stimulation (20 ng/mL). The protein level of p-P65 was detected by western blot (**d**) and the grayscale values are shown (**e**). The mRNA (**f**) and secretion (**g**) levels of sST2 were detected by qPCR and ELISA. Data represent mean ± SEM. *p < 0.05, **p < 0.01, ***p < 0.001 determined by student’s *t* test (**b**, **c**), one-way ANOVA (**e**) or two-way ANOVA (**f**, **g**).

Since sST2 production can be stimulated by TNF-α via activating NF-κB pathway [17], we further examined the influence of IL-37D transgene on NF-κB pathway in WAT from HFD-fed mice. We showed that IL-37Dtg mice subjected to HFD feeding had higher levels of IκBβ protein but lower levels of p-P65 protein in WAT than those in HFD-fed WT mice (Fig. 6a–c), indicating that IL-37D transgene could inhibit the activation of NF-κB pathway in WAT from HFD-fed mice. As HFD feeding may induce large amounts of TNF-α from WAT, which can simulate sST2 expression, we next treated WAT explants from NCD-fed WT mice with TNF-α in the presence or absence of recombinant human IL-37D protein. We found that TNF-α obviously induced the upregulation of p-P65 protein levels in WAT, while IL-37D protein significantly inhibited the upregulation (Fig. 6d, e), suggesting that exogenous IL-37D protein could inhibit TNF-α-induced NF-κB activation in WAT. In line with the suppression of NF-κB signaling, the increase of sST2 in response to TNF-α was dramatically inhibited by IL-37D protein on both mRNA and secretion levels (Fig. 6f, g), indicating that exogenous IL-37D could also block NF-κB activation and sST2 production in WAT.

**Fig. 7:**
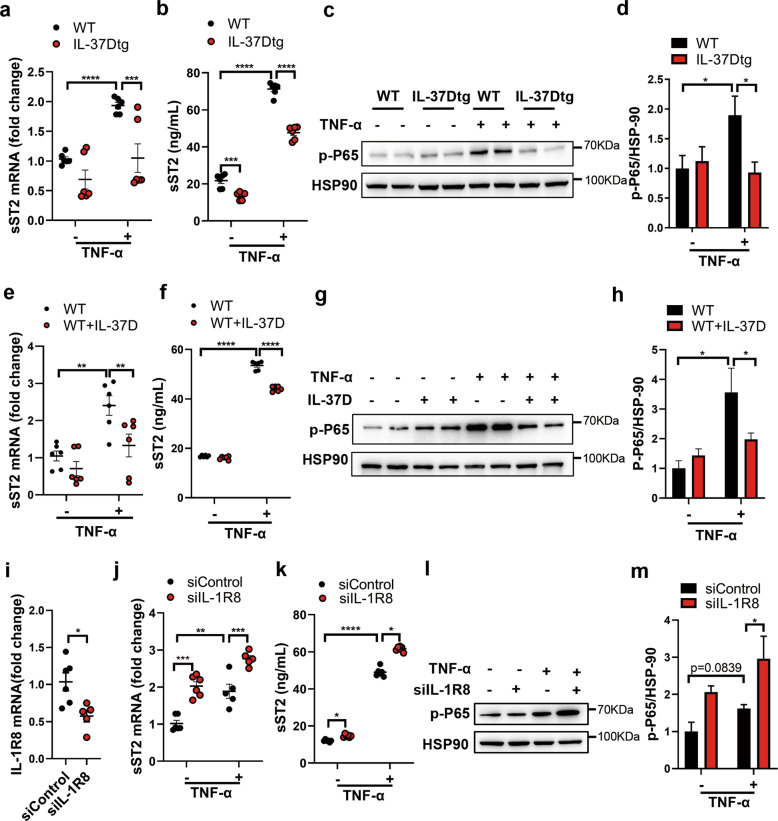
IL-37D inhibits sST2 production in IL-1R8-dependent manner. **a**–**d** ADSCs (1 × 10^5^/mL) from inguinal WAT of WT and IL-37Dtg mice were stimulated by TNF-α (10 ng/mL) for 24 h. The mRNA (**a**) and secretion (**b**) levels of sST2 were detected by qPCR and ELISA. The protein level of p-P65 was detected by western blot (**c**), the grayscale values are shown (**d**). **e**–**h** ADSCs from inguinal WAT of WT mice were treated with IL-37D protein (1 ng/mL) in the presence or absence of TNF-α (10 ng/mL) for 24 h. The mRNA (**e**) and secretion (**f**) levels of sST2 were detected by qPCR and ELISA. The protein level of p-P65 was detected by western blot (**g**) and the grayscale values are shown (**h**). IL-1R8 was silenced in ADSCs from IL-37Dtg mice, the mRNA levels of IL-1R8 (**i**) and sST2 (**j**) were detected by qPCR, the secretion level of sST2 was detected by ELISA (**k**), and the protein level of p-P65 was detected by western blot (**l**) and the grayscale values are shown (**m**). Data in **a**–**m** are representative of three independent experiments. Data represent mean ± SEM. *p < 0.05, **p < 0.01, ***p < 0.001, ****p < 0.0001 determined by two-way ANOVA (**a**, **b**, **d**–**f**, **h**, **j**, **k**, **m**) or student’s *t* test (**i**).

In addition to adipocytes, SVF also contributes to sST2 production from WAT [17], in which ADSCs account for large amounts of cellular components. Considering the dominant expression of IL-37D in human ADSCs [27], we checked the production of sST2 from ADSCs and the effects of IL-37D on its expression. In response to TNF-α, ADSCs from WT mice showed obvious sST2 elevation on both mRNA and secretion levels, while this upregulation was significantly decreased in those from IL-37Dtg mice, suggesting that IL-37D transgene could inhibit the expression and production of sST2 from ADSCs (Fig. 7a, b). Consistent with the impact of NF-κB activation on sST2 expression, TNF-α stimulation induced an obvious increase in the protein level of p-P65 in WT ADSCs rather than in IL-37Dtg ADSCs, supporting that IL-37D transgene could block TNF-α-induced NF-κB activation in ADSCs (Fig. 7c, d). To verify the exact pathway by which IL-37D inhibited sST2 production, we treated WT ADSCs with exogenous human IL-37D protein, and found similar results as ADSCs with endogenous expression of IL-37D. IL-37D protein significantly inhibited the increase of sST2 induced by TNF-α stimulation on both mRNA and secretion levels (Fig. 7e, f), whilst the upregulation of p-P65 protein levels was also inhibited by IL-37D protein (Fig. 7g, h). These observations support that both exogenous and endogenous IL-37D could inhibit NF-κB activation and sST2 expression in ADSCs. It is very likely that IL-37D functions through an extracellular membrane receptor pathway. IL-1R8 is verified to be associated with IL-37B and α chain of IL-18 receptor (IL-18Rα) to form the tripartite ligand-receptor complex on LPS-stimulated human mononuclear cells [21, 29]. To elucidate the possible influences of membrane receptor pathway on IL-37D effects, we further knocked down the expression of IL-1R8 in ADSCs from IL-37Dtg mice. IL-1R8 silence markedly abolished the inhibition on sST2 mRNA and protein by IL-37D transgene in ADSCs, even in the absence of TNF-α stimulation (Fig. 7i–k). Notably, IL-1R8 silence significantly increased the protein levels of p-P65 in IL-37Dtg ADSCs upon TNF-α stimulation (Fig. 7l, m), suggesting that IL-37D inhibited NF-κB activation relying on IL-1R8. These findings supported that IL-37D reduced sST2 production from ADSCs in an IL-1R8-dependent manner, by which IL-37D blocked NF-κB activation to inhibit sST2 expression.

## Discussion

Previously, we have demonstrated the protection of human IL-37D against endotoxemia and colitis in mouse models [27, 28]. In the present study, we reveal a novel function of human IL-37D in regulating type 2 immune homeostasis in WAT that facilitates WAT beiging and inflammation resolution, thereby providing protection against diet-induced obesity and associated metabolic disorders. In response to HFD feeding, mice with human IL-37D transgene showed a resistance to obesity that may directly contribute to their improvement of insulin action. In line with the relatively lean phenotypes, HFD-fed IL-37Dtg mice showed an increase in energy expenditure via thermogenesis compared with HFD-fed WT mice, which was further confirmed by UCP-1 upregulation in WAT together with reduced fat mass and adipocyte hypertrophy.

It has been recognized that type 2 immune cells such as ILC2s and M2 macrophages work alone or cooperate each other to promote beiging and reduce inflammation in WAT [7–9, 30–33]. To maintain type 2 immune circuit, IL-33/ST2 signaling plays a crucial role in activating or expanding ILC2s, Tregs, and M2 macrophages, which is mediated by the binding of IL-33 to ST2 on these cells [15, 34]. In spite of the indispensable role of IL-33, other factors regulating IL-33/ST2 signaling to maintain WAT homeostasis remain to be uncovered. In the present study, we demonstrate that IL-37D is a pivotal regulator in enhancing IL-33/ST2 signaling to boost type 2 immunity in WAT, thereby contributing to WAT beiging and inflammation resolution in response to HFD challenge. It is evident that IL-37D transgene led to an upregulation of ST2^+^ immune cell population, accompanied by M2 macrophage polarization and type 2 cytokines secretion in WAT from HFD-fed mice. Of note, sST2, a recently discovered adipokine induced by obesity [17], was blocked by IL-37D transgene in HFD-fed mice, indicating that sST2 may be a potential target for IL-37D to regulate IL-33/ST2 signaling. Indeed, sST2 treatment largely compromised the beneficial effects of IL-37D transgene, resulting in failure of its protection against obesity and metabolic disorders. These findings support that IL-37D acts as sST2 inhibitor to promote ST2 signaling-mediated type 2 immune homeostasis in WAT, thereby combating obesity and metabolic disorders.

Similar to the effects of IL-37D transgene, recombinant IL-37D protein also inhibited the expression and production of sST2 from WAT explants in response to TNF-α stimulation. Thus, IL-37D, either from exogenous administration or endogenous expression, may function in an extracellular signaling pathway. Indeed, when challenged with TNF-α, ADSCs had high ability to produce sST2, while both IL-37D transgene and IL-37D protein effectively inhibited TNF-α-induced NF-κB activation and sST2 production in these cells. Thus, ADSCs may act as another cell population apart from adipocytes that regulate sST2 and IL-33/ST2 signaling in WAT. Intriguingly, as the downstream proinflammatory cytokine of NF-κB pathway, the production of TNF-α from IL-37Dtg or IL-37D-treated ADSCs was also decreased, which in turn abolished its stimulation to NF-κB activation, further reduced sST2 production, thus forming a positive feedback loop to strengthen the effect of IL-37D on sST2 suppression. It has been demonstrated that IL-37B can associate with IL-18Rα and IL-1R8 to form a tripartite ligand-receptor complex on LPS-stimulated human peripheral blood mononuclear cells, thus dampening the inflammatory pathways including NF-κB signaling [21, 29, 35]. Our previous study also demonstrated that human IL-37D transgene inhibited NF-κB signaling dependent on IL-1R8 in mouse macrophages [28]. To our interest, IL-1R8 knockdown in IL-37Dtg ADSCs obviously abolished the suppression of IL-37D on NF-κB activation and sST2 production, demonstrating that IL-37D functions in an IL-1R8-depnednent manner to inhibit NF-κB activation and sST2 expression. In addition, NF-κB inhibition together with decreases in TNF-α and Srebp1c expression were also detected in liver tissues from HFD-fed IL-37Dtg mice compared with those from HFD-fed WT mice (Fig. S6a–c). Since TNF-α signaling can stimulate hepatic lipogenesis via promoting the expression or activation of Srebp1c [36–39], the decrease of TNF-α in liver tissues might relieve hepatic lipogenesis via weakening the role of Srebp1c, thereby attenuating the development of fatty liver in HFD-fed IL-37Dtg mice.

**Fig. 8:**
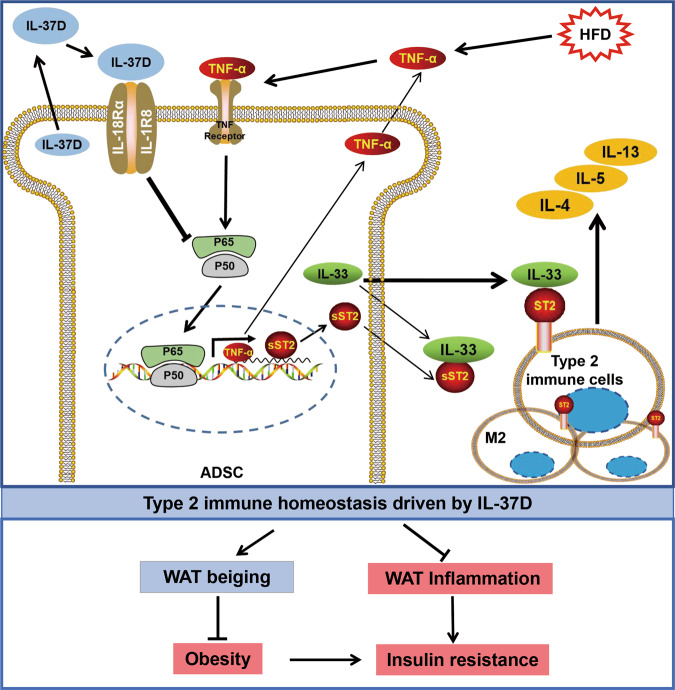
Working model for IL-37D-mediated sST2 inhibition in promoting WAT type 2 immune homeostasis. IL-37D inhibits sST2 production from ADSCs through IL-1R8-dependent NF-κB inactivation, which promotes IL-33/ST2 signaling to boost type 2 immune homeostasis in WAT characterized by amplification of ST2^+^ type immune cells and increase of type 2 cytokines, thereby providing protection against diet-induced obesity and insulin resistance through WAT beiging and inflammation resolution.

Taken together, this study provides evidence for the potential of human IL-37D to promote type 2 immune homeostasis in WAT by suppressing sST2, which is characterized by the amplification of ST2^+^ immune cells, M2 macrophage polarization, and type 2 cytokines secretion. The maintenance of type 2 response contributes to inflammation resolution and beiging in WAT, thereby providing protection against diet-induced obesity, insulin resistance, and fatty liver in HFD-fed IL-37Dtg mice (Fig. 8). Thus, human IL-37D may become a promising therapeutic target for obesity and associated metabolic diseases.

## Materials and methods

### Animals

C57BL/6 mice were provided by Vital River Laboratory Animal Technology (Beijing, China). IL-37Dtg mice were generated by Cyagen Biosciences (Suzhou, China) as previously described [27]. Male mice at the age of 8 weeks were fed on HFD (60% of total calories; Trophic Animal Feed High-tech, Nantong, China) for 18–23 weeks to induce obesity, using normal chow diet fed mice as lean controls. In some experiments, recombinant sST2 (R&D Systems, Emeryville, CA) was intraperitoneally administrated into IL-37Dtg mice (2 μg per mice every 3 days) 3 weeks before the end of HFD feeding. Animal studies were approved by Ethics Committee of Shandong University. All procedures were in accordance with the institutional guidelines for animal care and utilization.

### Metabolic study

Glucose tolerance test was performed in mice with an overnight fast. Insulin tolerance test was performed in mice with free access to food. The levels of blood glucose were measured at indicated timepoints before and after intraperitoneal injection of glucose (2 g/kg body weight; Sigma-Aldrich, Taufkirchen, Germany) or human insulin (0.75 units/kg body weight; Wanbang, Xuzhou, China). Energy expenditure, volumes of CO_2_ (VCO_2_) and O2 (VO_2_), and locomotor activity of the mice were measured using calorimetry system (TSE systems, Bad Homburg, Germany).

### Adipose tissue explant

WAT was cut into small pieces and cultured with M199 medium (0.5 g tissue/mL) in the presence of 1 nM insulin and 1 nM dexamethasone (Sigma-Aldrich, Taufkirchen, Germany) for 24 h. In some experiments, epididymal WAT explants (0.1 g tissue/mL) were stimulated with TNF-α (20 ng/mL; PeproTech, Rocky Hill, NJ) in the presence or absence of recombinant human IL-37D protein (10 ng/mL) for 24 h. The secretion levels of IL-4, IL-5, IL-13, sST2, and TNF-α were detected, the mRNA level of sST2 and protein level of p-P65 were examined.

### ADSC culture and treatment

Subcutaneous ADSCs were isolated from inguinal fat of the mice at the age of 10–12 weeks as previously described [40]. Briefly, fat pads were digested with 2 mg/mL collagenase I (Worthington, Lakewood, NJ) to isolate stromal vascular fraction cells. After overnight incubation, adherent cells were cultured as ADSCs for subsequent experiments. ADSCs were treated with TNF-α (10 ng/mL) for 24 h, the cells and supernatants were collected for assay. In some experiments, ADSCs were transfected with IL-1R8 siRNA (siIL-1R8; GenePharma, Shanghai, China) using INTERFERin (Polyplus, Illkirch, France) before TNF-α stimulation.

### Recombinant IL-37D preparation

Mature human *IL-37D* (21-197AA) cDNA was cloned into pET-22a plasmid and transformed into *E.coli. Rosetta*. The ultrasonic lysate of the bacteria was purified by using Ni resin (TAKARA, Osaka, Japan). Endotoxin was removed by EteraSeTM (Xiamen Bioendo Technology, Xiamen, China). The level of endotoxin in purified IL-37D was below 0.1 EU/mg, as determined by Chromogenic End-point Tachypleus Amebocyte Lysate (Xiamen Bioendo Technology, Xiamen, China).

### Hepatic triglyceride detection

Liver tissues were weighed and homogenized. After centrifugation, the supernatants were measured for triglyceride using commercial kit (JianCheng Bioengineering Institute, Nanjing, China). Frozen sections of liver tissues were stained with Oil Red O solution (Servicebio, Wuhan, China) followed by hematoxylin staining.

### ELISA

Serum levels of insulin were measured with mouse insulin ELISA kit (Crystal chem, Elk grove village, IL). The secretion levels of sST2, TNF-α, IL-4, IL-5 and IL-13 were measured using mouse sST2 ELISA kit (R&D Systems, Emeryville, CA), mouse TNF-α ELISA kit (Biolegend, San Diego, CA), and mouse IL-4, IL-5, IL-13 ELISA kit (Elabscience, Wuhan, China), respectively.

### Quantitative PCR

Total RNA was extracted with TRNzol reagent according to the manufacturer’s instruction (Tiangen, Beijing, China), and reversely transcribed into cDNA with PrimeScript™ RT reagent Kit with gDNA Eraser (TAKARA, Osaka Japan). Quantitative PCR was performed using FastStart Universal SYBR Green Master (Roche Applied Science, Penzberg, Germany). Primer sequences for indicated genes are shown in Table S1.

### Western blot assay

Proteins from lysed cells or tissues were separated on SDS-PAGE gel and transferred onto PVDF membranes. After blocked with 5% bovine serum albumin, the membranes were probed overnight at 4 °C with primary antibodies against UCP-1 (Abcam, Cambridge, England), p-P65, IkBβ (CST, Boston, MA), HSP90 (ZSGB-BIO, Beijing, China), β-actin or β-tubulin (Proteintech, Wuhan, China) followed by HRP-conjugated secondary antibodies (Jackson ImmunoResearch; West Grove, PA) for 1 h at room temperature. Then membranes were visualized by ECL detection system.

### Flow cytometry

The SVF of WAT was incubated with Fc blocker (Biolegend) and then stained with fluorochrome-conjugated antibodies against CD11b (M1/70), CD45 (30-F11), ST2 (DIH9), CD206 (C068C2), CD11c (N418) (Biolegend), and F4/80 (T45-2342) (BD Biosciences, San Jose, CA) or corresponding isotypic antibodies in dark at 4 °C for 30 min. Dead cells were excluded by staining with Zombie UV Fixable Viability dye (Biolegend). Data were acquired on Aria III (BD Biosciences) or CytoFLEX (Beckman Coulter, Brea, CA) and analyzed with FlowJo software (Tree Star) or CytExpert (Beckman Coulter).

### Statistical analyses

The sample sizes were described in the figure legend. No exclusions of data were made that would significantly impact the results or conclusions. Male mice with the same genotype and similar age were randomly assigned to experimental groups. Investigators were not blinded during the experiment. Data were analyzed by student’s *t* test, one-way ANOVA, or two-way ANOVA using the GraphPad Prism 8 (GraphPad Software, San Diego, CA). Data represent the mean ± SEM, *P* < 0.05 was considered significant.

## Supplementary information

Supplemental Material

## Data Availability

All data needed to support the conclusions of this article are present in the article and its supplement information files.
