# Supplementary material for: IL-37 isoform D acts as an inhibitor of soluble ST2 to boost type 2 immune homeostasis in white adipose tissue
Source: Cell Death Discov. 2022 Apr 5;8:163. doi: 10.1038/s41420-022-00960-3 (PMC8983676; doi:10.1038/s41420-022-00960-3)
Supplement: Supplementary file 1 — Supplemental Material [file 41420_2022_960_MOESM1_ESM.docx]

**Supplemental Material**

**Table S1 Sequences for qPCR primers and siRNA.**

| **Gene** | **Sequence (5'-3')** |
| --- | --- |
| murine *Srebp1* | F-GGAGCCATGGATTGCACATT  R-GGCCCGGGAAGTCACTGT |
| murine *Tnfα* | F-CCCTCACACTCAGATCATCTTCT  R-GCTACGACGTGGGCTACAG |
| murine *Il6* | F-CTGCAAGAGACTTCCATCCAG  R-AGTGGTATAGACAGGTCTGTTGG |
| murine *Il1β* | F-ACCTTCCAGGATGAGGACATGA  R-AACGTCACACACCAGCAGGTTA |
| murine *Il1r8* | F-GTGACATGGCCCCTAATTTCC  R-ATGCCAGACCATCTTTCAGCC |
| murine *sST2* | F-TCGAAATGAAAGTTCCAGCA  R-TGTGTGAGGGACACTCCTTAC |
| murine *Il33* | F-TCCAACTCCAAGATTTCCCCG  R-CATGCAGTAGACATGGCAGAA |
| murine *Gapdh* | F-AGGTCGGTGTGAACGGATTTG  R-TGTAGACCATGTAGTTGAGGTCA |
| human *IL37D* | F-TGAACCCCAGTGCTGCTTAG  R-CCCAGAGTCCAGGACCAGTA |
| *siIL-1R8* | GAUACAAACUCUUCCUAGATT |
| *siControl* | UUCUCCGAACGUGUCACGU |


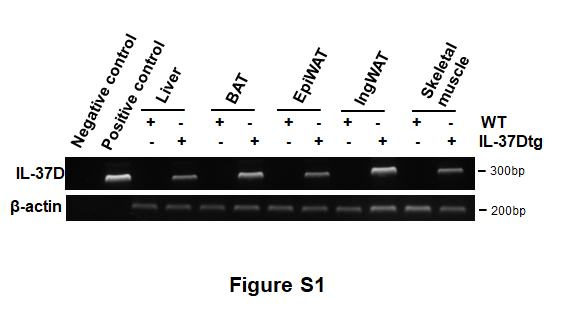

**Figure S1.** The mRNA expression profiles of human IL-37D were determined in tissues from IL-37Dtg or WT mice by PCR.


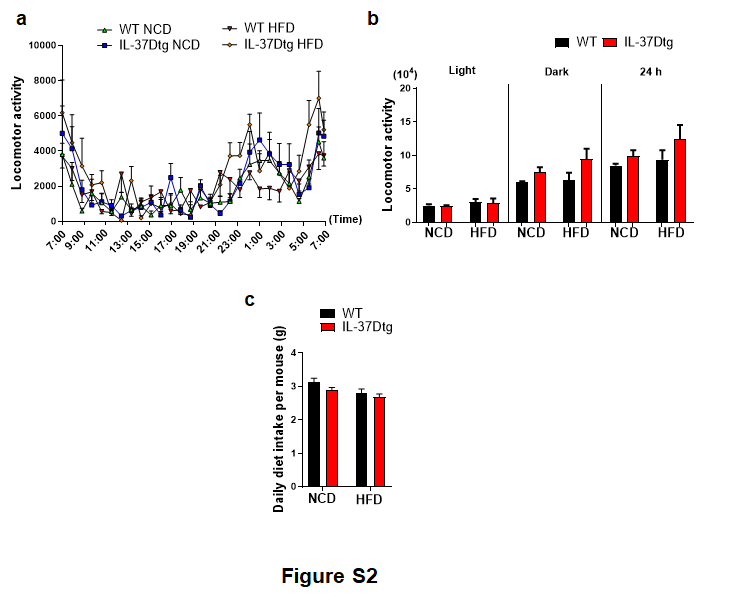


**Figure S2.** The locomotor activity (**a, b**) and diet intake (**c**) were determined in IL-37Dtg and WT mice (n=4 per group). Data represent mean ± SEM, determined by two-way ANOVA (**a-c**).


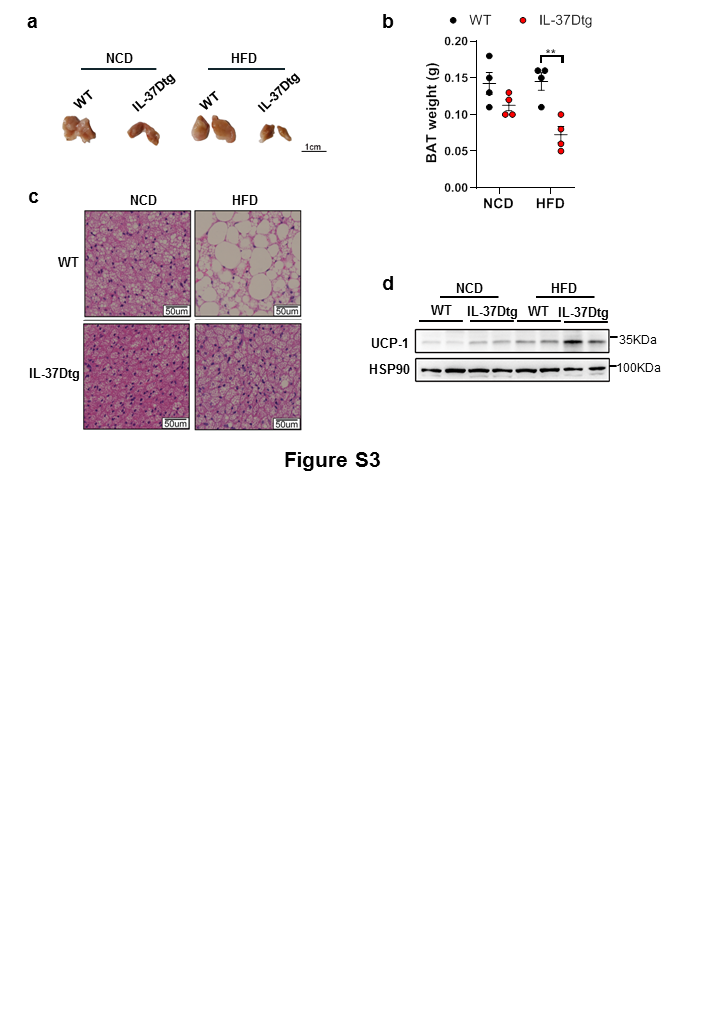


**Figure S3.** IL-37Dtg and WT mice were fed on 18 weeks of HFD (n=4 per group). The morphology (**a**) and weight (**b**) of BAT are shown. The sections of BAT were stained with H&E (**c**). The protein level of UCP-1 was detected by western blot (**d**). Data represent mean ± SEM.**p< 0.01, determined by two-way ANOVA (**b**).


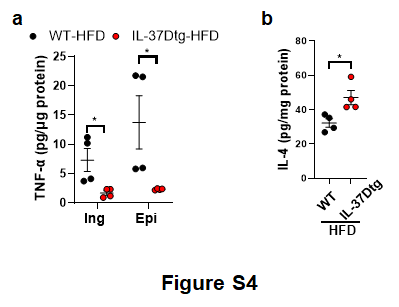


**Figure S4**. Mice were fed with HFD for 23 weeks (n=4 per group). The secretion levels of TNF-α (**a**) in WAT explants were determined by ELISA. The secretion level of IL-4 (**b**) from epidydimal WAT explants was determined by ELISA. Data represent mean ± SEM. *p < 0.05, determined by student’s *t* test (**a, b**).


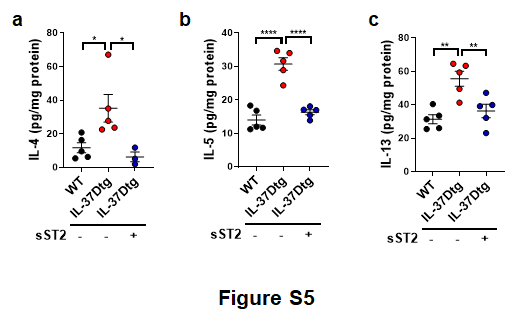


**Figure S5**. IL-37Dtg and WT mice were fed on HFD for 16 weeks (n=5 per group), recombinant sST2 was intraperitoneally injected into IL-37Dtg mice for 3 weeks (2μg every 3 days). The secretion levels of IL-4 (**a**), IL-5 (**b**), IL-13 (**c**) from epidydimal WAT explants were detected by ELISA. Data represent mean ± SEM. *p< 0.05, **p< 0.01, ****p< 0.0001 determined by one-way ANOVA (**a-c**).

**
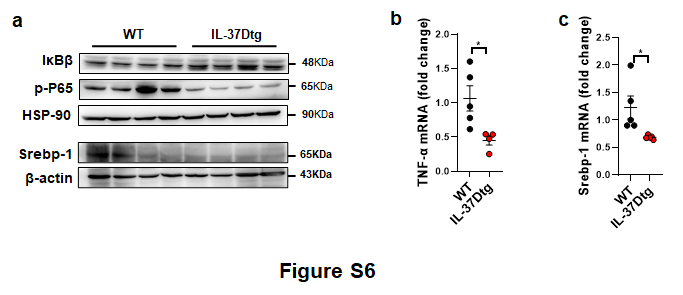
**

**Figure S6**. Mice were fed with HFD for 23 weeks (n=4 per group). The protein levels of p-P65, IκBβ and Srebp-1 in liver were determined by western blot (**a**). The mRNA levels of TNF-α (**b**) and Srebp-1 (**c**) in liver tissues were determined by qPCR. Data represent mean ± SEM. *p < 0.05, determined by student’s *t* test (**b, c**).
